# Benefits of Whey Proteins on Type 2 Diabetes Mellitus Parameters and Prevention of Cardiovascular Diseases

**DOI:** 10.3390/nu15051294

**Published:** 2023-03-06

**Authors:** Jean-François Lesgards

**Affiliations:** Ingénierie des Peptides Thérapeutiques, Ambrilia-Cellpep, Faculté de Médecine Nord, Aix-Marseille University, Boulevard Pierre Dramard, 13015 Marseille, France; jf.lesgards@gmail.com; Tel.: +33-0650684562

**Keywords:** whey proteins, type 2 diabetes mellitus, postprandial hyperglycemia, gut hormones, satiety, antioxidant

## Abstract

Type 2 diabetes mellitus (T2DM) is a major cause of morbidity and mortality, and it is a major risk factor for the early onset of cardiovascular diseases (CVDs). More than genetics, food, physical activity, walkability, and air pollution are lifestyle factors, which have the greatest impact on T2DM. Certain diets have been shown to be associated with lower T2DM and cardiovascular risk. Diminishing added sugar and processed fats and increasing antioxidant-rich vegetable and fruit intake has often been highlighted, as in the Mediterranean diet. However, less is known about the interest of proteins in low-fat dairy and whey in particular, which have great potential to improve T2DM and could be used safely as a part of a multi-target strategy. This review discusses all the biochemical and clinical aspects of the benefits of high-quality whey, which is now considered a functional food, for prevention and improvement of T2DM and CVDs by insulin- and non-insulin-dependent mechanisms.

## 1. Introduction

Cardiovascular diseases (CVDs) are the major cause of death globally. Around 17.9 million people died from CVDs in 2019, representing 32% of all deaths [1]. Type 2 diabetes mellitus (T2DM) is a major comorbidity associated with CVDs. Globally, overall CVDs affect around 32.2% of all people with T2DM [2], and CVDs are a major cause of mortality among people with T2DM, accounting for approximately half of all deaths.

Global diabetes is a pandemic with a prevalence in 20–79-year-olds in 2021 of 10.5% (536.6 million people), which should reach 12.2% (783.2 million) in 2045 [3]. Individuals with T2DM are at high risk for microvascular complications (including retinopathy, nephropathy, and neuropathy) as well as macrovascular issues (cardiovascular comorbidities) caused by hyperglycemia and insulin resistance [4]. Diabetes and hypertension share similar risk factors, such as dyslipidemia, obesity, endothelial dysfunction, and vascular inflammation leading to atherosclerosis.

In general population, diabetes and CVDs are linked to various parameters in particular diet, physical activity, walkability, and air pollution (NO_2_ and PM 2.5) more than genetics [5,6,7].

Diet is a crucial factor to prevent diabetes and CVDs, and patients with T2DM may benefit from diminishing added sugars and increasing proteins and non-processed as well as vegetable fats [8,9,10]. We know this in particular, as decades of added sugar damage to health have been largely underestimated in comparison with “bad fats”, such as cholesterol or saturated fat, whose harmful effects have been pointed out excessively worldwide. We now know that the Sugar Research Foundation (SRF) since the 1950s has constantly paid millions of U.S. dollars to feed people with false information based on warped or false studies [11]. This has been only recently been clearly denounced according to precise documents from the SFR in the *Journal of the American Medical Association* (JAMA) [12] and has been reproduced in the New York Times [13]. The real “bad fats” that have a clear negative impact on diabetes and CVDs are essentially processed fats and trans-fats but not necessarily saturated fats [14].

Meta-analyses suggest that certain food interventions and diets such as the Mediterranean diet have a beneficial role on CVD prevention in populations, including individuals with diabetes [15,16]. Red meat and processed meats seem to be associated with T2DM, whereas soy and milk products seem to protect against T2DM. Egg and fish intake do not appear to be significantly associated with a decrease in T2DM risk [17].

Dairy products, in particular low-fat dairy, are negatively associated with T2DM [18]. Milk protein is made primarily of whey and casein proteins, representing approximately 20 and 80% of the total proteins, respectively [19].

Whey, a by-product of cheese and curd manufacturing, was formerly considered a waste product. It is a protein complex derived from milk, which is nowadays presented as a functional food, having a number of health benefits [20]. The biological components of whey include β-lactoglobulin (β-Lg) (45–57%), α-lactalbumin (α-La) (15–25%), immunoglobulins (IGs) (10–15%), glycomacropeptide (up to 15%), serum albumin (SA) (10%), lactoferrin (Lf) (≈1%), and lactoperoxidase (<1%). Interestingly, Hippocrates already praised the health benefits of whey in Ancient Greece, and it was used as a medicine through the Middle Age (for burn soothing, vitality, and to improve various illnesses) [21]. There are diverse types of whey protein products available for nutrition and food supplements: whey protein concentrates (WPCs) (which contain 25%–80% protein), whey protein isolates (WPIs) (containing ≥ 90% protein), whey protein hydrolysates, β–Lg, α-La, and protein-peptone fraction [22].

The goal of this review is to discuss all the biochemical and clinical aspects of the benefits of whey for prevention and improvement of postprandial hyperglycemia, T2DM, and CVDs.

## 2. Whey and Dairy Product Intake Induce Benefits on T2DM Parameters

In a meta-analysis on cohort studies, Tong et al. reported that T2DM risk could be decreased by 5% with dairy products and 10% for low-fat dairy products with daily intakes [18]. Another meta-analysis of seventeen cohort studies demonstrated significant inverse associations between T2DM risk and intake of total dairy products and low-fat dairy products [23].

### 2.1. Whey Intake Improved Insulin Secretion and Postprandial Glycemia

Milk-derived whey as well as casein proteins can produce insulin secretion in obese, pre-diabetic, and also type 2 diabetes individuals [24,25,26,27,28,29]. Studies in humans have shown that whey protein decreases postprandial glycemia and could be used in medical/nutritional therapy to regulate blood sugar [30,31]. In diabetic subjects, whey intake has been associated with a reduction of postprandial hyperglycemia [32,33]. Even a small 15 g dose of whey protein consumed shortly before mixed-macronutrient meals stimulates insulin release, improves postprandial glycemia (−13%), and increases satiety in T2DM subjects (*p* < 0.05) [34].

Various studies have similarly reported positive effects of whey protein on insulin secretion [35]. The intake of 50 g WPI associated with maltodextrin increased insulin production by 96% versus maltodextrin alone in pre-diabetic adults (*p* < 0.05) and a 21% decrease in postprandial blood glucose after protein meals (*p* < 0.0001) [36]. Interestingly, for practical nutrition, the addition of whey (27.6 g) to high-glycemic-index meals (such as bread and mashed potatoes and meatballs) increases insulin release (31% for breakfast and 57% for lunch, both *p* < 0.05) and diminishes postprandial blood glucose (−21%, *p* < 0.05) excursion in type 2 diabetic subjects [37].

In lean and healthy subjects, whey consumption has also been shown to decrease blood sugar [36,38]. Between whey, tuna, turkey, and egg albumin, the measure of postprandial glucose and insulin concentrations in 22 lean, healthy men provided the best results for whey [24]. Blood glucose was significantly lower for whey meal than for turkey (*p* < 0.023) and eggs (*p* < 0.001), indicating a faster glucose uptake in cells, but not with the tuna meal. Blood insulin was also significantly higher for whey compared to tuna, turkey, and eggs (all *p* < 0.001).

Moreover, whey protein may have beneficial effects on some symptoms of metabolic syndrome and improve cardiovascular risk factors [35,39,40]. Metabolic syndrome is a combination of hyperglycemia, hypertension, excess body fat around the waist, and abnormal cholesterol or triglyceride levels, increasing the risk of diabetes, heart disease, and stroke [41]. Moreover, a meta-analysis of 22 randomized controlled trials (RCTs), using the Cochrane method for the elimination of bias, showed that whey intake decreased insulin significantly in patients with metabolic syndrome (weighted mean difference (WMD): −0.94; 95% CI: −1.68, −0.21) but did not have an effect on fasting plasma glucose levels [42]. Meanwhile subgroup analyses showed a significant reduction in fasting plasma glucose levels and other meta-analyses including in obese participants who had also shown improvement in fasting plasma glucose levels after whey protein intake [43,44].

### 2.2. Effects on Insulin Resistance and Glycated Hemoglobin

A meta-analysis of 22 randomized controlled trials (RCTs) highlighted a significant decrease in glycated hemoglobin (HbA1c) with whey intake in patients with metabolic syndrome (WMD: −0.15; 95% CI: −0.29, −0.01) and in the Homeostatic Model Assessment of Insulin Resistance (HOMA-IR) (WMD: −0.20; 95% CI: −0.36, −0.05) [42]. Another meta-analysis of 30 RCTs suggested that dairy intake, in particular, low-fat dairy products, has a positive action on HOMA-IR (mean difference (MD) of −1.21; 95% CI −1.74 to −0.67; *p* < 0.00001; *I*^2^ = 92%) [45].

Dairy protein consumption before a meal decreases food intake and, in association with carbohydrates, decreases glycemia by insulin-dependent as well as insulin-independent mechanisms [46,47].

Interestingly, whey can also be effective for controlling blood sugar parameters and inflammation before surgery. Fasting before surgery, which can be prolonged from 10 up to 16 h, can induce hyperglycemia due to a limitation of insulin action by the effect of counter-regulatory hormone action. Whey protein in a drink associated with carbohydrates has been shown to minimize the postoperative insulin resistance (HOMA-IR) and associated acute inflammation vs. carbohydrates alone (2.75 ± 0.72 vs. 5.74 ± 1.16; *p* < 0.05) [48].

## 3. Mechanisms of Whey and Dairy Proteins Associated with Decrease in Postprandial Glycemia

### 3.1. Activity of Amino Acids on Insulin Secretion

It has been confirmed that the insulinotropic effect of dairy proteins is associated with certain amino acids, in particular the branched-chain amino acids (BCAAs) who seem to be of vital importance, especially leucine, isoleucine, valine, lysine, and threonine, inducing insulin secretion with leucine, reportedly having the greatest effect acutely [49,50,51]. Leucine activates glutamate dehydrogenase activity in β-cells, which leads to an increase in Krebs cycle activity, oxygen consumption by these cells, and then to increased insulin production [49]. Leucine and high protein intake also seem to modulate AMP-activated protein kinase (AMPK) and mTOR and influence hypothalamic neuropeptides, reducing the expression of orexigenic neuropeptides (NPY) and AgRP (Agouti-related peptide) and increasing anorexigenic neuropeptide pro-opiomelanocortin (POMC) [51].

**Figure 1:**
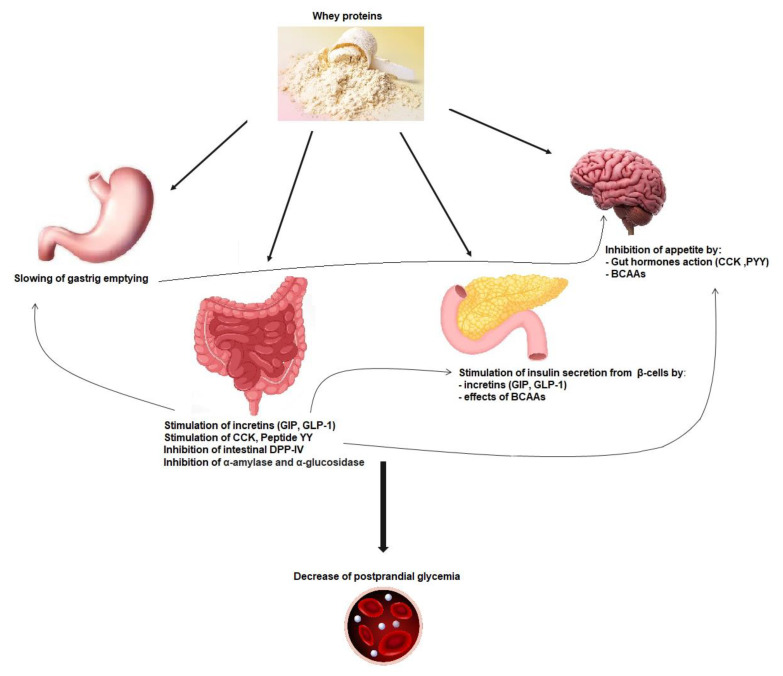
Mechanisms implicated in whey protein activity on postprandial glycemia reduction. GIP: glucose-dependent insulinotropic polypeptide; GLP-1: glucagon-like-peptide-1; CCK: cholecystokinin; PYY: peptide YY; DPP-IV: dipeptidyl peptidase-IV; BCAAs: branched-chain amino acids.

Whey protein is an exceptional source of BCAAs, which are easily and quickly digested, leading to a rapid increase in BCAA leads in the circulation and insulin release, which may improve postprandial hyperglycemia (Figure 1) [42]. Glutamate and alanine can also participate in insulin secretion coupling, not alone but by amplifying the stimulation by glucose [50]. Cysteine could also be implicated in this process [52].

### 3.2. Incretin Secretion and Insulin Secretion

Dairy-protein-derived peptides can also increase the insulin secretion effect through dipeptidyl peptidase-4 (DPP-4) inhibitory activity in the proximal gut, preventing the incretin glucose-dependent insulinotropic polypeptide (GIP) and glucagon-like-peptide-1 (GLP-1) degradation [40] (Figure 1) [53]. Indeed a major part of secreted insulin is a result of the action of both GIP and GLP-1. However, because of the cleaving activity of DPP-4, GIP and GLP-1 have a half-life of only 1–2 min [54]. Thus, inhibiting DPP-IV activity is considered as a way of treatment in T2DM [55]. Incretins also increase the sensitivity of β-cells to glucose, stimulate β-cell proliferation, and protect these cells against apoptosis [56].

When provided shortly prior to a meal, whey dose-dependently reduces (using from 9 to 18 g) the postprandial glycemia (*p* < 0.0001) and increases GLP-1 levels (*p* < 0.0001) [57]. Bioactive substances in whey, among which are IGs, Lf, α-La, and glutamine, have been shown to increase incretin hormones and to inhibit dipeptidyl peptidase-IV [31,42,58,59]. Other studies have shown a strong increase in GLP-1 concentrations after a whey drink when compared to a glucose or fructose drink (from 25 to 50 g of whey, *p* < 0.05) taken before a meal (30 min to 4 h) [60,61].

Some bioactive peptides should be responsible for influencing postprandial incretin responses [62]. Indeed, whey and milk proteins are degraded during low-pH digestion in the stomach and by gastric pepsin and other peptidases. The resulting hydrolyzed proteins pass into the small intestine and are further split by pancreatic proteases into single amino acids and oligopeptides and finally by other enzymes from the brush-border enzymes into dipeptides, tripeptides, and amino acids. Bioactive peptides can contain from two to twenty amino acid residues or more. Some of these peptides have been identified in the gastrointestinal tract as well as in bloodstream after milk intake, but more studies are needed to fully characterize these peptides and their precise role in glycemia management [62].

However, in present hypertriglyceridemia, obesity and high-baseline GLP-1 levels tend to have poorer response to whey proteins [63], and even positive results have been also observed [25]. It could be linked to glucagon-induced increase with whey protein intake [60]. Furthermore, hypertriglyceridemia augments the hyperglycemic effects of glucagon [64].

Whey proteins seem also to inhibit other enzymes such as α-amylase and α-glucosidase [53,65,66].

### 3.3. Gastric Emptying Effect on Postprandial Glycemia

The actions of whey proteins on gastric emptying, on postprandial glycemia, and on secretion of incretin hormones are linked together. In addition to its impact on insulinotropic effects, GLP-1 induced by whey intake is also able to slow down gastric emptying by relaxing the proximal stomach, reducing antral and duodenal motility, and increasing pyloric tone. This restrains energy intake and can inhibit glucagon secretion, which all together improve postprandial glycaemia (Figure 1) [67]. The function of the gastrointestinal tract is key for glucose homeostasis, especially during the postprandial phase, and slowing gastric emptying can diminish postprandial glycemic excursions in healthy and diabetic subjects (Figure 1) [58,68,69,70]. Other gut hormones, namely, cholecystokinin (CCK) and peptide YY (PYY), can decrease gastric emptying and appetite [71].

The importance of slowing gastric emptying is key in the decrease in postprandial glycemia observed when proteins are added to glucose intake [72]. Likewise, a whey “preload” is able to slow gastric emptying of a following meal in both healthy [73] and T2DM subjects [74]. In diabetic subjects, GLP-1 was higher when whey was ingested (55 g) between −15 min and 90 min before the meal versus during the mean (*p* < 0.001), even if the incremental area under the curve (iAUC) was not significantly different [74], and both decrease postprandial glucose (363.7 ± 64.5 mmol · min^−1^ · L^−1^) and (406.3 ± 85.9 mmol · min^−1^ · L^−1^) compared with no whey (734.9 ± 98.9 mmol · min^−1^ · L^−1^; *p* < 0.005).

### 3.4. Gut Hormones, Amino Acids, and Satiety

Satiety induced by proteins has been demonstrated, in an acute manner, with meals containing from 25 to 90% proteins, leading to a significant decrease in energy intake. It has also been shown with high protein content in ad libitum diets, lasting from a few days up to 6 months [75]. Among the three macronutrients, protein has the greatest satiating action. After protein-containing nutrient intake, signals can be sent to the central nervous system (CNS) via gastric and gut peptide action and via the bloodstream after digestion. Indeed, satiety is induced by various mechanisms, which are both visceral (during digestion) and metabolic (inter-prandial phase) and directed towards the CNS directly at the level of the hypothalamus and indirectly mainly through the vagus nerve [76]. Regarding meal size, the negative feedback control from gastrointestinal signals and bloodstream takes place in the dorsal vagal complex (brainstem) and in the hypothalamus.

The gut peptide hormones upregulated by whey consumption include CCK, PYY, GLP-1, and GPI (Figure 1) [56,58,73]. It has been proposed that high protein meals could induce the greatest production of PYY and the highest satiety feeling in obese as well as normal-weight human subjects [74]. Ghrelin, an orexigenic peptide decreased after consumption of proteins; leptin; and insulin levels are also known to influence satiety [58,60,74].

Although GLP-1 can have an action on peripheral organs through the circulation, it is of note also that GLP-1 can be produced by the pancreas and brain as well [56].

Studies demonstrate that dairy and whey proteins decrease appetite better than other protein sources such as eggs, casein, or soy [73,77,78,79]. In the study from Hall et al., plasma CCK was increased by 60% (iAUC, *p* < 0.005), GLP-1 by 65% (iAUC, *p* < 0.05), and GIP by 36% (iAUC, *p* < 0.01) following a 48 g whey preload when compared with casein, showing the particular potential of whey in this field [73]. Whey, tuna, turkey, and egg albumin meals were compared in terms of appetite measures and energy intake in 22 lean healthy men. The best results were obtained for whey, with a significant reduction of mean energy intake at the ad libitum meal 4 h after (*p* < 0.001) [24]. Appetite rated by the subjects, postprandial insulin, and energy intake during the meal were strongly related.

## 4. Other Mechanisms of Whey and Dairy Proteins Associated with the Benefits on T2DM and Cardiovascular Risk

### 4.1. Effects of Whey Intake on Lipid Profile

Different works have shown that whey consumption ameliorates some biological markers in obesity and T2DM such as fasting lipids.

In a study with whey supplementation in overweight/obese individuals (27 men and women with a mean age of 48 years) for 12 weeks, fasting triglycerides were lowered after 6 weeks (*p* < 0.025) as well as a total cholesterol and LDL cholesterol after 12 weeks in the whey group (*p* < 0.001 and 0.003, respectively) [25]. It was also lowered in comparison with a casein group (*p* < 0.026 and *p* < 0.045, respectively). In this study, fasting insulin measurements and insulin resistance were also significantly diminished in the whey group versus the control (*p* < 0.049 and *p* < 0.034, respectively).

A systematic review and meta-analysis of 22 RCTs in patients with metabolic syndrome, using the Cochrane Collaboration risk of bias tool, showed a significant decrease in the values for triglycerides (WMD: −17.12; 95% CI: −26.52, −7.72), total cholesterol (WMD: −10.88; 95% CI −18.60, −3.17), LDL-cholesterol levels (WMD: −8.47 95% CI: −16.59, −0.36), and total cholesterol/HDL cholesterol ratio (WMD: −0.26; 95% CI: −0.41, −0.10) [42]. Another meta-analysis of 13 trials showed that whey protein supplementation significantly decreased blood triglyceride level by 0.11 mmol/L (95% CI: −0.21, 0 mmol/L), but had no effects on blood total cholesterol (−0.11 mmol/L, 95% CI: −0.27, 0.05 mmol/L), LDL- cholesterol levels (−0.08 mmol/L, 95% CI: −0.23, 0.07 mmol/L), and HDL-cholesterol (0.01 mmol/L, 95% CI: −0.04, 0.05 mmol/L) [80]. Other encouraging results were highlighted in a meta-analysis including nine RCTs, among which, trials on overweight and obese patients and whey intake improved HDL-cholesterol and total cholesterol levels as well as systolic and diastolic blood pressure and fasting plasma glucose (all *p* < 0.05) [43]. Moreover, in metabolic syndrome, in subjects receiving yogurt enriched with whey (5 g), calcium (500 mg), vitamin D (500 IU), prebiotic fiber (3 g), and probiotic cultures for 10 weeks, a significant reduction of triglyceride levels (*p* < 0.001) and insulin resistance (HOMA-IR) (*p* = 0.025), as well as increased HDL-cholesterol levels (*p* < 0.01), were observed [81].

Altogether, these results indicate that whey intake could improve lipidemia. It has been proposed that protein intake could decrease the production of chylomicrons [40]. The insulinotropic effect of whey proteins may enhance lipoprotein lipase (the enzyme that hydrolyses triglycerides in chylomicrons and very low density lipoprotein (VLDL)) activity and increase the clearance of chylomicrons [29]. Whey proteins can also decrease the intestinal absorption of cholesterol as well as its de novo generation in the liver [25].

### 4.2. Improvement of Obesity and Weight by Whey Intake

Decreasing weight and obesity can improve diabetes parameters and prevent CVDs. A double-blind RCT on 90 obese subjects for 23 weeks, comparing whey protein to soy (≈56 g/d of protein and 1670 kJ/d) and to an isoenergetic carbohydrate intake, showed significant decreases of body weight and fat mass of 1.8 kg (*p* < 0.006) and 2.3 kg (*p* < 0.005), respectively, in comparison with the carbohydrate group [82]. Body weight and composition did not differ significantly between the groups consuming soy and whey or between soy and carbohydrates. No lean mass difference was observed between all groups. Moreover, waist circumference decreased significantly in the whey group versus the two others (*p* < 0.05). This is supported by a meta-analysis on 30 RCTs, which suggests that low-fat dairy products may have beneficial results on waist circumference in a total of 1348 individuals (MD: −1.09 cm (95% CI 1.68 to −0.58; *p* < 0.00001; *I*^2^ = 94%)) and body weight in 2362 individuals with 0.42 kg less than control (*p* < 0.00001; *I*^2^ = 92%) [45]. In another meta-analysis with nine RCTs included, whey intake led to a significant decrease in body weight (MD = 0.56, 95% CI: 0.30–0.81), lean mass (MD = 0.77, 95% CI: 0.59–0.96), and fat mass (MD = 1.12, 95% CI: 0.77–1.47) [43].

Furthermore, whey has also been shown to decrease fat in the liver of obese patients [83].

People with less protein in their diet have more chances of developing obesity, such as in women and children [84,85,86]. Clinical studies and meta-analyses show that whey decreases fat mass, total weight, and waist circumference when sugar is lowered and replaced by protein in isoenergetic meals in overweight/obese people after a few weeks/months [44,82,87].

Whey proteins and proteins in general also have an interesting metabolic property that can be useful for losing weight and avoiding obesity: digesting and processing proteins requires more energy/calories than lipids and fats for the body [88], called the “thermic effect of food” (TEF). Evidence suggests TEF as a weight-loss tool for research and clinical studies [89]. Indeed, reported TEF for protein is 20–30% of energy content, being 0–3% for fats and 5–10% for carbohydrates [90,91].

Moreover, if a diet for weight loss is only based on dietary energy restriction (500–750 kcal/d energy deficit), the problem is that 25% of the body mass that is lost is muscle/lean mass [86]. Then, high-quality protein is needed to avoid this lean mass loss [84,92]. Whey protein is one of the best quality proteins with regard to its amino acid profile (high essential, branched-chain, and leucine content) and fast digestibility [93]. Whey protein has been found to stimulate muscle protein synthesis with a significantly better yield than casein, soy, and other proteins, and clinical studies have confirmed the interest of whey to avoid losing muscle/lean mass [94,95,96]. Meta-analyses observing shorter-term as well as long-term studies on energy restriction point out that the daily protein intake necessary to avoid lean mass loss would be between 1.2 and 1.5 g protein/kg/d (i.e., ≈89–119 g protein/d for women and 104–138 g protein/d for men) [92,97,98,99]. However, other works suggest that lower quantities (i.e., 0.8–1.2 g protein/kg/d) seem to be enough for the preservation of lean mass during an energy restriction diet [100].

### 4.3. Effect of Whey Intake on Hypertension

High blood pressure/hypertension is a major global public health issue. Hypertension and diabetes T2DM are interrelated diseases that strongly predispose to CVDs and stroke [101].

Increasingly more clinical studies have shown that milk, fermented milk products, and peptides from whey and casein could bring about significant improvement in blood pressure [102]. Some studies demonstrated that whey intake ameliorates blood pressure in overweight and obese individuals. In a study with whey protein or glucose (27 g) supplementation for 12 weeks (70 subjects), systolic blood pressure was significantly lower with whey after 6 weeks (*p* < 0.05) as well as diastolic blood pressure (*p* < 0.05) after 12 weeks [25]. Moreover, an improvement of vascular function was observed as measured by a decreased augmentation index (AI), which is an indirect measure of arterial stiffness, after 12 weeks of whey intake (*p* < 0.05).

Potent reductions in systolic blood pressure have been observed with several whey peptides containing four or fewer amino acid residues [103]. The short peptides described by FitzGerald et al. are YGLF f(50–53) in *α*-La, IPA f(78–80) in β-Lg, FP f(221–222) in bovine serum albumin, and GKP f(18–20) in *β*2-microglobulin, which produced maximum decreases in systolic blood pressure of 23, 31, 27, and 26 mmHg, respectively.

### 4.4. Importance of Antioxidant and Anti-Inflammatory Potential of Whey Proteins with Regard to T2DM and Cardiovascular Health

Whey protein can exert antioxidative effects, in the first place, as an enhancer of the synthesis of reduced glutathione (GSH) and can also activate the endogenous antioxidative enzyme system. The correlation between low glutathione (GSH) and diabetes has been described. Diabetes has been linked to oxidative damage and decreased GSH content (also increased GSSG/GSH ratio, oxidized glutathione/reduced glutathione) in different tissues [104,105,106]. The decrease in GSH is, in most cases, associated with an increased activity of NF-kB, an inflammation node [107], and GSH is key in lowering oxidative stress and insulin resistance [52,108].

Several studies have highlighted that in obese patients, oxidative stress is also associated with a decrease in GSH levels [109,110] and a decrease in the GSH/GSSG ratio [111]. Furthermore, nutritional stress caused by a diet high in fats and carbohydrates promotes oxidative stress, as evidenced by increased lipid peroxidation products, decreased antioxidant system, and lower GSH levels [112,113].

If physical activity can lead to an improvement of the antioxidant systems and defenses as well as reduced risks of CVDs in overweight populations, the synergy of resistance exercises in association with whey intake has been shown to be more efficient [114].

Antioxidant/anti-inflammatory capacities of whey and dairy have been shown in various conditions with both low- and full-fat dairy products, as well as fermented dairy foods, even if some studies showed no effect [115,116,117,118]. Moreover, a few studies have already shown the interest of whey in other conditions as in gut inflammation and Crohn’s disease [119,120], leaky gut [121,122], or rheumatoid arthritis [123].

## 5. Discussion on Long-Term Effects of Whey Protein and of Other Natural Compounds on Glycemic Parameters, T2DM, and Cardiovascular Health

Consuming a 15 g premeal of whey protein before each main meal decreases daily hyperglycemia by 8% (*p* < 0.05), which helps to maintain the body for longer periods (≈2 h/day, *p* < 0.05) in an euglycemic area, especially for people with T2DM [32]. Although most of the recent evidence in systematic reviews and meta-analysis show beneficial effects of whey protein supplementation on postprandial and short-term glycemic response, as well as blood lipid profile, other long-term clinical data are needed for better understanding the benefits of whey intake on postprandial and baseline glycemia after several weeks/months [42,124,125,126]. Increasingly more studies have investigated the effects of whey and its bioactive peptides and biochemical and biological pathways, especially on longer periods on glucose and lipid metabolism, hypertension, oxidative stress and inflammation, and vascular health [127,128]. Some studies suggest that regular whey intake may positively affect long-term glycemic control [129,130]. Whey may also consolidate arterial walls, which can prevent and improve cardiovascular diseases [25,59]. Altogether, these results indicate a potential for whey in preventing and improving pre-diabetic and diabetic conditions, as well as hypertension and cardiovascular diseases.

Moreover, of importance, the best whey products, WPC and WPI, are concentrates obtained by microfiltration at low temperature (cold-process) and without applying extreme pH (acid or basic) as is it used in the “ion exchange” process, in order to protect the most sensitive amino acids such as sulfur amino acids, tryptophan, and others. Meanwhile, most of the whey encountered on the market are processed with heat and/or with strong acids or bases, both of which can denature the 3D structure of proteins and oxidize some key amino acids, which have strong benefits for our health [131,132,133].

Indeed, whey proteins can be denatured by heat, inducing the stabilization of disulfide bonds, gelation, and possible loss of a part of antioxidant activity [22,132,133]. Heat-treated WPC solutions can generate these disulfide-bonded aggregates between all proteins present in whey: β-Lg, α–La, IgG, SA, and Lf [131]. Heat stability of the major whey protein is relative to globular proteins (β-Lg and α-La), and complete denaturation may occur at 90 °C beyond 10 min [134]. For minor whey proteins, i.e., SA and IGs, denaturation starts at ≈65 °C, whereas for the major whey proteins, denaturation starts only above 70–75 °C [135,136]. Thus, the order of sensitivity to heat-induced denaturation is the following: IGs > SA > β-Lg A > β-Lg B > α-La [135,136,137]. This denaturation can increase the oxidability of various sensitive amino acids. With heat, milk and whey proteins can be altered by Maillard reactions [138]. Among the several types of amino acids, the sulfur amino acids are more prone to oxidation. Methionine, for example, is very easily oxidized, producing methionine sulfoxide as a first step, which has been found in heated milk, whey, and casein products [139,140].

Other natural products and foods than dairy and whey are known to have the potential to prevent or improve T2DM. Studies and meta-analyses suggest that certain food interventions and diets such as the Mediterranean diet in particular, rich in fruits and vegetables, nuts, beans, cereals, and fish rather than red meat, and unsaturated oils and fats such as olive oil, have a beneficial role in CVDs, prevention in populations including people with T2DM [15,16,141]. An amount of 200 g/day of fruit intake seems to be a threshold to reduce the risk of T2DM [142,143], and it is linked with their rich content in dietary polyphenols, which may also decrease T2DM risk and associated complications, although some controversial results have been also published [144,145]. Some alkaloids such as berberine, trigonelline, and capsaicin are also promising compounds that may be useful in the treatment of T2DM through various mechanisms including the inhibition of α-glucosidase and DPP-IV and modulation of oxidative stress [146].

Moreover, natural sugars present in plants such as iminosugars (monosaccharide sugars in which nitrogen replaces the ring oxygen) that can inhibit gut α-glucosidase and reduce carbohydrate breakdown in the upper gastrointestinal tract [147,148,149]. Some medicines have already been developed from this research such as Glyset^®^ and Zavesca^®^, which are derived from the natural compound 1-deoxynojirimycin [147]. Meanwhile, mainly at the level of the digestive system, undigested saccharides become a food source for microbial fermentation [150].

Since diabetes is a multifactorial condition, it is not surprising that there is still no simple cure or drug for managing blood glucose levels, T2DM, and other cardiovascular comorbidities. The main drugs are biguanides, such as metformin, which reduce gluconeogenesis in the liver; sulfonylureas, which stimulate insulin secretion; thiazolidinediones, which are insulin sensitizers; and various other tracks, among which are aldose reductase and tyrosine phosphatase 1B, free fatty acid receptor 1 (FFAR1), G-protein-coupled receptor (GPCR), peroxisome-proliferator-activated receptor-γ (PPARγ), sodium glucose co-transporter-2 (SGLT2), α-glucosidase, aldose reductase, glycogen phosphorylase (GP) [151]. In the future, efficient strategies against T2DM should focus on multi-target compounds [152]. This is where natural compounds such as whey, which has various action pathways, seem useful and promising and would deserve longer-term studies (months, years) to assess their efficacy on various T2DM and glycemia parameters. The magnitude of hyperglycemia reduction induced by whey has been found to be comparable with some interventions with drugs such as sulfonylureas, and several authors conclude that this should have implications for nutritional strategies against T2DM [74]. Whey could be associated with other natural compounds such as polyphenols, alkaloids, or iminosugars.

Furthermore, metformin and several drugs that have been developed have various side effects (mild and serious) that are also responsible for a lack of adherence and compliance from the subjects [153]. On the contrary, whey is a very safe nutrient/supplement. For nutrition interventions, whey should be used to adjust the protein intake in order to reach the global daily needs between 0.8 and 1.2 g/kg body weight/day, which is necessary for optimization of body functions, but probably not higher, even though there is no evidence that too much protein can damage the kidneys of healthy people [154]. High-protein meals are not advised for subjects with renal issues as they can lead to glomerular hyper-filtration, raise the pressure inside the kidneys, and accelerate chronic kidney diseases [155,156].

Finally, new axes could emerge more strongly in T2DM research, such as the impact of intestinal microbiota on the regulation of insulin content, insulin resistance, and the regulation of blood glucose [157]. Various pathological pathways may be implicated in the process, through gut barrier health, inflammation, levels of incretins, or the production of short-chain fatty acids (SCFAs). Several measures based on intestinal flora, diets, and supplements such as probiotics and others could be used to treat and even prevent T2DM [158]. Various studies have already shown the benefits of whey with strong prebiotic effect on the gut microbiota on normal weight as well as and obese subjects with increase in *Bifidobacterium* and *Lactobacillus* groups [159] and on gut inflammation, Crohn’s disease [119,120], and leaky gut improvement [121,122].

Moreover, the impact of whey intake, alone or in association with polyphenol compounds for example, on the improvement of antioxidant/anti-inflammatory status as well as the increase in the time passed in euglycemia state (also associated with less oxidative stress [160]) are areas that deserve to be studied for T2DM management.

Thus, together with healthy diets, without forgetting other key parameters such as physical activity and avoiding too high air pollution [5], high-quality whey can be a valuable tool for managing postprandial hyperglycemia and associated oxidative stress, blood lipid profile, and insulin resistance, and it can globally contribute to prevention and improvement of T2DM and CVDs, even if more long-term studies are needed.

## Data Availability

Not applicable.
